# Todd Paralysis in Rolandic Epilepsy

**DOI:** 10.15844/pedneurbriefs-29-7-1

**Published:** 2015-07

**Authors:** Pasquale Striano, Maria Stella Vari

**Affiliations:** 1 Pediatric Neurology and Muscular Diseases Unit, Department of Neurosciences, Rehabilitation, Ophthalmology, Genetics, Maternal and Child Health University of Genoa, “G. Gaslini” Institute, Genova, Italy

**Keywords:** Post-Ictal Paresis, Rolandic Epilepsy, Migraine

## Abstract

Investigators from University of Gaziantep, Turkey described the clinical and EEG findings of patients with benign epilepsy of childhood with centrotemporal spikes (BECTS) experiencing postictal Todd paralysis.

Investigators from University of Gaziantep, Turkey described the clinical and EEG findings of patients with benign epilepsy of childhood with centrotemporal spikes (BECTS) experiencing postictal Todd paralysis. The study was conducted at the Division of Pediatric Neurology, Gaziantep University. The authors investigated a total of 108 BECTS children, aged 2-16 years, between 2011 and 2014. Detailed information regarding patient's clinical manifestations, seizure duration, and postictal features were collected. A 125-item questionnaire including diagnostic criteria from the International Classification of Headache Disorders, 2nd edition, was administered.

Overall, 12 patients (11% of the total) experienced postictal transient motor deficits, i.e., Todd paralysis. There were 6 boys and 6 girls, with average age of 8.08 years. Statistical analysis failed to find any difference between children with or without postictal paralysis, including seizure semiology or duration and EEG findings.

In the large majority of the patients (11 out of 12) Todd paralysis occurred only once in their life and followed a focal motor seizure involving predominantly the upper extremity and the face. There was significant difference (p <.0001) in the incidence of migraine in patients who did not have Todd paralysis (13/96, 13.5%) compared to patients who experienced Todd paralysis (10/12, 83.3%). All migraine patients were successfully treated with anticonvulsants, usually carbamazepine or levetiracetam. [1]

COMMENTARY. BECTS is one of the most frequent epileptic syndromes in children [2]. Migraine is strongly comorbid in RE. Prevalence of migraine in BECTS probands is 15% versus 7% in nonepilepsy probands, and in siblings of RE probands prevalence was 14% versus 4% in nonepilepsy siblings [3], suggesting shared susceptibility to migraine and rolandic epilepsy that is not directly mediated by epileptic seizures. In addition, epilepsy and migraine frequently show a clinical overlap and children with migraine frequently show EEG abnormalities, including rolandic discharges [4]. Moreover, migraine is commonly associated with other epilepsy syndromes, including other forms of idiopathic childhood epilepsies, e.g., Panayiotopoulos syndrome [2]. Despite the fact that migraine and epilepsy are clearly distinct disorders, it has been suggested that they could share some pathophysiologic mechanisms and clinical manifestations. Furthermore, it is likely that any of triggering factors, irrespective of their nature (genetically determined or not), could potentially lead to a paroxysmal and transient cortical excitability change leading to prolonged neuronal depolarization (seizure) or spreading depression (migraine) [5].

In summary, the present study confirms that there is a comorbidity of migraine and rolandic epilepsy and those children who experience postictal Todd paralysis are more likely to have migraine [1]. The reasons for this finding are unclear. Nevertheless, the awareness of this post-ictal phenomenon and its spontaneous resolution within hours from the onset is crucial in the paediatric setting as early diagnosis may prevent unneeded hospitalization and further investigations, as well as helping to reduce parents’ concerns.
